# Response of the Green Alga *Chlamydomonas reinhardtii* to the DNA Damaging Agent Zeocin

**DOI:** 10.3390/cells8070735

**Published:** 2019-07-17

**Authors:** Mária Čížková, Monika Slavková, Milada Vítová, Vilém Zachleder, Kateřina Bišová

**Affiliations:** Laboratory of Cell Cycles of Algae, Centre Algatech, Institute of Microbiology, Czech Academy of Sciences, 37981 Třeboň, Czech Republic

**Keywords:** DNA damage, double-stranded break, zeocin, caffeine, cell cycle, cyclin-dependent kinase, *Chlamydomonas reinhardtii*

## Abstract

DNA damage is a ubiquitous threat endangering DNA integrity in all living organisms. Responses to DNA damage include, among others, induction of DNA repair and blocking of cell cycle progression in order to prevent transmission of damaged DNA to daughter cells. Here, we tested the effect of the antibiotic zeocin, inducing double stranded DNA breaks, on the cell cycle of synchronized cultures of the green alga *Chlamydomonas reinhardtii*. After zeocin application, DNA replication partially occurred but nuclear and cellular divisions were completely blocked. Application of zeocin combined with caffeine, known to alleviate DNA checkpoints, decreased cell viability significantly. This was probably caused by a partial overcoming of the cell cycle progression block in such cells, leading to aberrant cell divisions. The cell cycle block was accompanied by high steady state levels of mitotic cyclin-dependent kinase activity. The data indicate that DNA damage response in *C. reinhardtii* is connected to the cell cycle block, accompanied by increased and stabilized mitotic cyclin-dependent kinase activity.

## 1. Introduction

All living organisms are exposed to numerous DNA damaging agents, including ionizing and ultra-violet (UV) irradiation, various chemicals, and cell metabolites. Therefore, it is vital for the cell to continuously check the integrity of genetic material and to repair it immediately after damage. Mechanisms responsible for the detection and repair of damaged DNA are organized into a sophisticated protein network—the DNA damage response pathway that activates DNA repair, blocks further progression through the cell cycle to prevent spreading of the damage to daughter cells or, in the case of extreme DNA damage, initiates the cell death pathway. One of the best characterized types of DNA damage, double-stranded breaks, can arise by exposing cells to free oxygen radicals generated either by normal metabolic processes or by exposure to external ionizing irradiation [1]. The primary detection of double-stranded breaks is conserved from yeast to mammals and plants; it involves recruitment and activation of ataxia telangiectasia mutated (ATM) kinase [1,2,3,4,5,6,7]. The pathway downstream of ATM and its relative ATR (ATM- and Rad3-related) are distinct in mammals and in plants. In mammals, the downstream targets involve checkpoint kinases 1/2 (Chk1/2) and protein p53, ultimately leading to activation of DNA repair, a block in cell cycle progression, or alternatively cell death [2,8]. Homologs of Chk1/2 and protein p53 are missing in the plant kingdom, and instead, DNA damage response in higher plants use a plant-specific transcription factor suppressor of gamma irradiation 1 (SOG1) [9] that is directly phosphorylated by ATM [10]. SOG1 shares many target genes with p53 as it is responsible for transcriptional induction of DNA repair and DNA replication genes, as well as cell cycle regulators but it also has specific targets among plant defense genes [11,12]. Moreover, it targets three MYB3R transcription factor repressors that are crucial for strong down-regulation of G2/M cell cycle-regulated genes [12].

Blocking of cell cycle progression in response to DNA damage, at the DNA damage checkpoint, is crucial for preventing the spread of DNA damage to daughter cells and thus for maintaining cell viability [2,3,8]. The DNA damage response varies in different phases of the cell cycle as there are different DNA templates and different sets of DNA repair proteins available [2,8]. In principle, there are at least two types of DNA damage checkpoints, one responding to DNA damage in S phase, another one responding to DNA damage during G2 phase, and possibly yet another one reacting to DNA damage during G1 phase [13]. The DNA damage checkpoint exploits the available cell cycle regulatory machinery, i.e., cyclin-dependent kinases, cyclins and their regulators. In plants, it seems to be at least two-pronged: 1) ATM (and ATR) through SOG1 transcription factor [11,12] induce two types of CDK inhibitors, Wee1 kinase [11] and SIAMESE RELATED CDK inhibitors [14], both of which will lead to CDK inhibition [13,15]. Furthermore, SOG1 controls proteasome-dependent degradation of the mitotic CDKB2;1 [16]. 2) In addition, SOG1 promotes accumulation of repressor type MYB3R transcription factors [17] leading to suppression of G2/M specific genes [18] (for a recent review on the topic see [15]).

**Figure 1:**
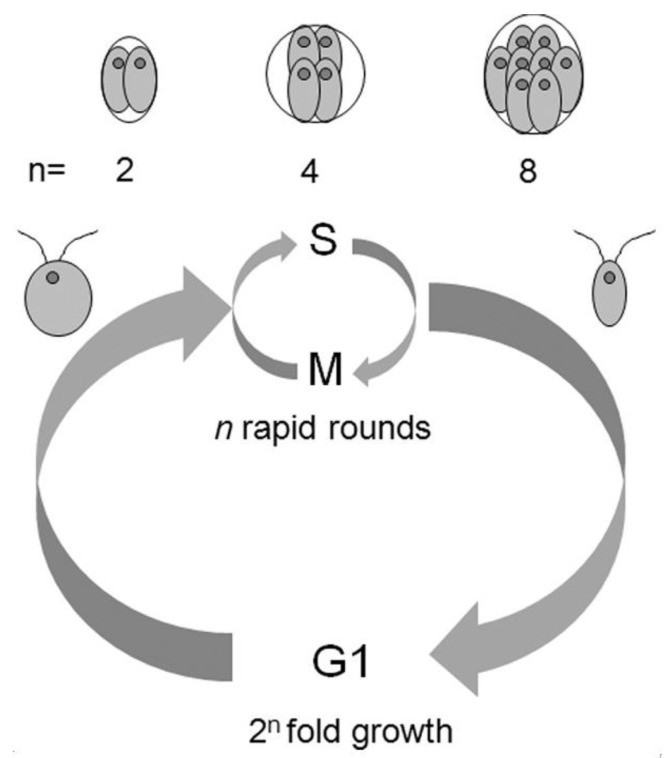
Schema of the *C. reinhardtii* cell cycle. A very long G1 phase is followed by a series of *n* rapid alternating rounds of S and M phase yielding 2^n^ daughter cells. The value of *n* is related to the extent of growth during the G1 phase. Divided mother cells with different numbers of divisions (*n*) and daughter cells produced are schematically drawn on the top (modified after [29]).

*Chlamydomonas reinhardtii* is a widely used model alga that divides by a mechanism described as multiple fission. Its cell cycle consists of a long G1 phase followed by *n* alternating rounds of S phase and mitosis, which are terminated by cell division into 2^n^ daughter cells (Figure 1; [19,20,21,22,23]). The number of daughter cells is dictated by the cell size [22,24]. During growth in G1 phase, cells consecutively attain one or several commitment points (CPs), each of them dependent upon reaching a critical cell size [22,24,25,26]. The attainment of CP is a prerequisite for one round of DNA replication, nuclear and cellular division [27,28], which occur independent of a further energy supply, i.e., even in the dark. The *C. reinhardtii* genome seems to lack not only homologs of Chk1/2 and p53 present in mammals but not in plants, but also homologs of plant specific Sog1 transcription factor, although it retains homologs of ATM/ATR. How they respond to DNA damage, particularly how they coordinate the DNA damage response with cell cycle progression, remains enigmatic.

Here, we have analyzed the behavior of *C. reinhardtii* cells in response to double-stranded DNA breaks mediated by the antibiotic zeocin. To understand how DNA damage response is connected with cell cycle progression, i.e., to gain an insight into the DNA damage checkpoint, we also analyzed activities of key cell cycle regulators, CDKs. The prolonged cell cycle block was maintained in the presence of persistent high mitotic kinase activity. This was in contrast to the response of *C. reinhardtii* cells to UV irradiation where kinase activity was kept low.

## 2. Materials and Methods

### 2.1. Experimental Organism, Culture Growth Conditions, Cell Cycle Synchronization and Analysis

*Chlamydomonas reinhardtii* (wild type CC-125) was obtained from the Chlamydomonas Resource Center at University of Minnesota (St. Paul, MN, USA). The cultures were grown on high salt (HS) nutrient medium as described by Sueoka [30] with double the concentration of Ca^2+^ ions and a tenfold increase in the Mg^2+^ ion concentration. Trace elements (1 mL per 1 L of medium) as described by [31] were used instead of the Hutner’s trace elements. For routine sub-culturing, the cultures were streaked onto modified HS medium solidified with agar every three weeks and grown on the light shelf at an incident light intensity of 100 µmol·m^−2^·s^−1^ of photosynthetically active radiation.

For the sensitivity tests, 50 mL of liquid HS medium were inoculated directly from plates into custom-made tubes (inner diameter 30 mm, height 190 mm, volume 50 mL). The tubes were illuminated by fluorescent tubes (OSRAM DULUX L55W/950 Daylight, Milano, Italy); the incident light intensity at the surface of the culture vessels was 500 µmol·m^−2^·s^−1^ of photosynthetically active radiation. The cultures were grown for 2 days in liquid at 30 °C, aerated with 2% CO_2_. When the cultures reached a cell density of 1 × 10^6^, they were serially diluted in HS medium, spotted onto HS plates containing different concentrations of zeocin and/or caffeine and left to grow on the light shelf for about a week.

For the synchronization experiments, cells were grown for at least three cycles of alternating 11 h light and 13 h dark periods (11 h / 13 h) at 30 °C in custom-made 300 mL tubes (inner diameter 30 mm, height 500 mm, volume 300 mL) in HS nutrient medium aerated with 2% (*v/v*) CO_2_; the light conditions were the same as described above. The cell concentration during the entire synchronization procedure was kept under 2 × 10^6^ cells/mL by dilution. For the experiment, the synchronized culture of daughter cells was diluted to a starting concentration of 1 × 10^6^ cells/mL and split into four plan-parallel 2.5 L vessels (440 × 245 × 23 mm, volume 2200 mL) and cultivated at the same temperature and light conditions. Each experiment was repeated at least three times. Individual processes of the cell cycle were performed in the same time window with the midpoints varying by a maximum of one hour.

Caffeine (Sigma-Aldrich spol. s.r.o. Prague, Czech Republic) was added to the specified final concentration from a 100 mM stock solution. Zeocin (Life Technologies Czech Republic s.r.o., Prague, Czech Republic) was added from a 100 mg/mL stock solution.

### 2.2. Cell Cycle Analyses

Attainment of the commitment point was evaluated according to Hlavová and colleagues [32] in aliquots taken hourly. The 10 mL aliquots were aerated at 30 °C in the dark until hour 10 of the cell cycle and then 1 mL aliquots of the culture were spread on HSM plates and incubated in darkness at 30 °C until cell division in the slowest dividing culture was complete. The plates were evaluated directly by microscopy. For each sample, the number of large single undivided mother cells and mother cells divided into 2, 4 or 8 daughter cells were counted (at least 150 mother cells total), and the percentage of each category was calculated and plotted as a function of time. The mother and daughter cells are easily distinguishable due to differences in cell size. Cell clumps can be distinguished thanks to the specific morphology of divided mother cells.

Cell division was assessed in hourly taken aliquots, fixed with glutaraldehyde (0.2% final concentration), and viewed under the light microscope. The percentage of cells undivided or divided into two, four or eight daughter cells were calculated and plotted as a function of time.

The cell volume was measured by a Multisizer 3 (Beckman Coulter Česká republika s. r. o., Prague, Czech Republic) in samples fixed with glutaraldehyde (0.2% final concentration) by diluting 50 μL of fixed cells into 10 mL of 0.9% NaCl; at least 200 cells were gated at the median size.

Total nucleic acids were extracted according to Wanka [33], as modified by Decallonne and Weyns [34]. The DNA assay was carried out as described by Decallonne and Weyns [35], with the modifications of Zachleder [36] (see also [37]).

### 2.3. Microscopy

Observations in transmitted light and fluorescence microscopy were carried out using an Olympus BX51 microscope (Olympus Czech Group, s.r.o., Prague, Czech Republic) equipped with a CCD camera (DP72). A U-MWIBA2 filter block (Ex/Em: 460-490/510-550 nm) was used for SYBR Green I fluorescence (Life Technologies Czech Republic s.r.o., Prague, Czech Republic).

For electron microscopy, the samples were fixed with glutaraldehyde (0.2% final concentration) in phosphate buffer (160 mM Na_2_HPO_4_, 40 mM KH_2_PO_4_, pH 7.2), post-fixed in osmium tetroxide, dehydrated and embedded in LR White resin. Ultrathin sections were picked up on nickel grids and double-stained with uranyl acetate and lead citrate, then examined using a TEM JEOL 1010 electron microscope (JEOL USA, Inc., Peabody, MA, USA).

### 2.4. Kinase Activity Assay

Cell pellets containing 2 × 10^7^ cells were harvested during the cell cycle, washed with SCE buffer (100 mM sodium citrate, 2.7 mM EDTA-Na_2_, pH 7 (citric acid)), snap frozen in liquid nitrogen and stored at –70 °C. The protein lysates were prepared and affinity purified by CrCKS1 beads as described [29]. Histone H1 kinase activity was assayed as previously [38] in a final volume of 10 µL with the CrCKS1 beads fraction corresponding to 20 µL of whole cell lysate. The reactions were started by adding the master mix to a final composition of 20 mM HEPES, pH 7.5, 15 mM MgCl_2_, 5 mM EGTA, 1 mM DTT, 0.1 mM ATP, 0.2% (*w/v*) histone (Sigma H5505) and 0.370 MBq [γ ^32^P] ATP.

Proteins were separated on 15% SDS-PAGE gels [39]. Phosphorylated histone bands were visualized by autoradiography. The extent of phosphorylation was quantified using Image Studio Lite software (LI-COR Biosciences, Lincoln, NE, USA) as described previously [40]. Each experiment was repeated at least four times and representative experimental results are shown.

## 3. Results

### 3.1. Caffeine Hypersensitizes Cells to DNA Damage

**Figure 2:**
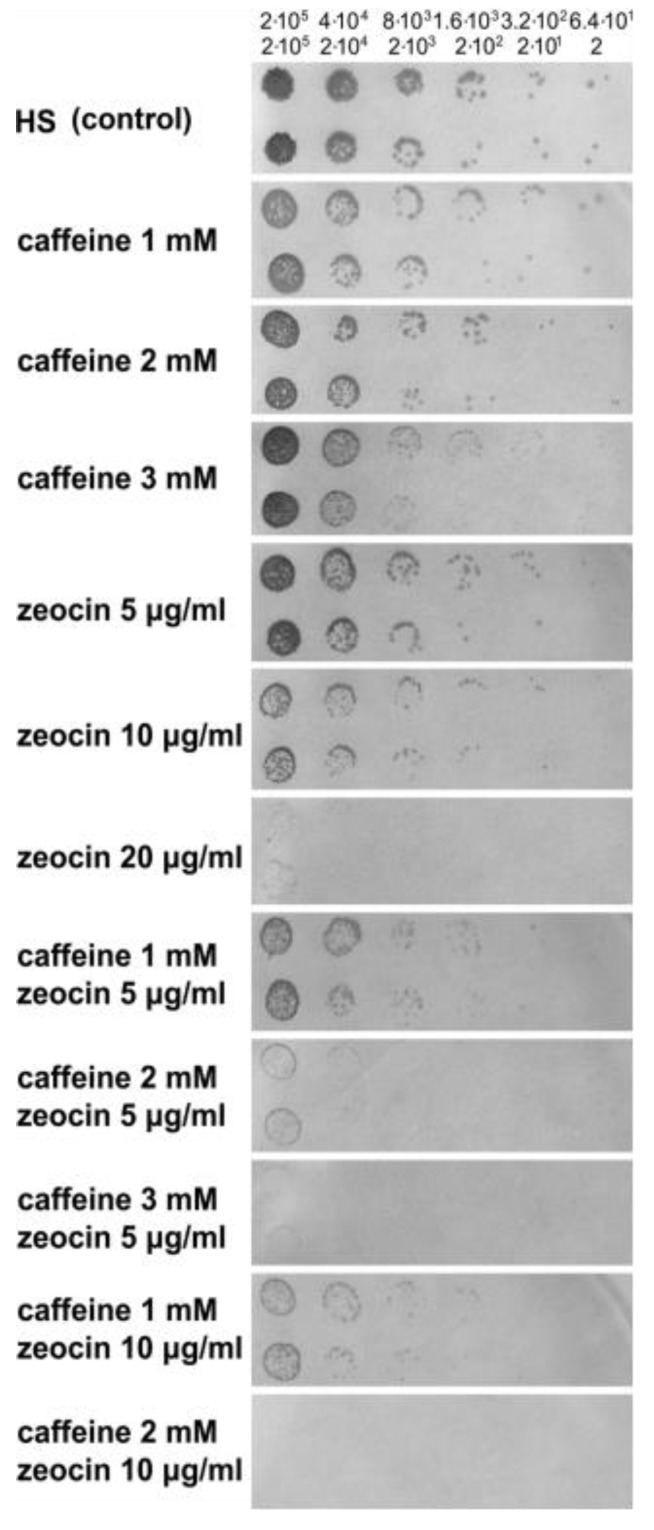
Survival of *C. reinhardtii* cells on caffeine and zeocin. Plate assay with serially diluted cells spotted either on HS nutrient medium free of chemicals (HS), used as a control, or onto HS nutrient medium supplemented with different concentrations of caffeine and zeocin. Two different serial dilutions are shown; upper strip—dilution by a factor of five, bottom strip—dilution by a factor of 10, approximate concentrations of the cells in each spot are indicated above; concentrations of the drugs are indicated at the side of each strip.

Zeocin is a bleomycin family antibiotic causing DNA damage by cleaving both strands of the DNA molecule [41,42,43]. Its application in different concentrations represents a simple way of administering a discrete amount of DNA damage to cells. Caffeine is known to be synergistic with many DNA damaging agents [44] in various organisms due to specific inhibition of ATM/ATR kinases [45,46,47]. To determine the concentrations of caffeine and zeocin affecting growth and survival of *C. reinhardtii*, wild type cells were serially diluted by a factor of ten or five, and then spotted onto HS nutrient medium containing different concentrations of caffeine, zeocin, or a combination of both (Figure 2). To assess the growth difference, the presence or absence of growing cells in each spot was visually compared to the control cultures grown on HS nutrient medium in the absence of chemicals. More subtle differences could be derived by comparing the number of colonies formed from single cells in individual spots. In general, increasing concentrations of caffeine affected cell survival only slightly when compared to increasing concentrations of zeocin. Caffeine, up to 2 mM, had virtually no effect. On the contrary, increasing zeocin concentrations severely affected cell survival due to DNA damage caused by the antibiotic. The combination of caffeine and zeocin was much more effective than any of the compounds alone (Figure 2). For experiments with synchronized cell cultures, concentrations of caffeine (2 mM) and zeocin (5 μg/mL) were chosen that had no or a negligible effect if applied alone but decreased cell survival to below 1% when combined.

### 3.2. Growth is Not Affected by Caffeine or Zeocin

To understand the details of absence of growth and decreased survival in the presence of zeocin, *C. reinhardtii* cultures were synchronized by three consecutive light/dark (11 h / 13 h) cycles to obtain a uniformed culture of daughter cells at the beginning of the light period and to be able to discriminate the effects on growth from those on cell cycle progression. Uniformity of the cultures was verified microscopically and by measuring cell size. Cultures used for experiments were made up of a population of cells, the size of which lay within the 95% confidence interval of 10 µm^3^. For the experiment, the same light/dark cycle was used in order to prevent further growth during the nuclear/cell division. Caffeine (2 mM), zeocin (5 μg/mL), or both agents were added to the treated cultures at 8 h into the light period, e.g., in late G1 phase before the DNA replication started and the cells entered the alternating rounds of S/M phase.

**Figure 3:**
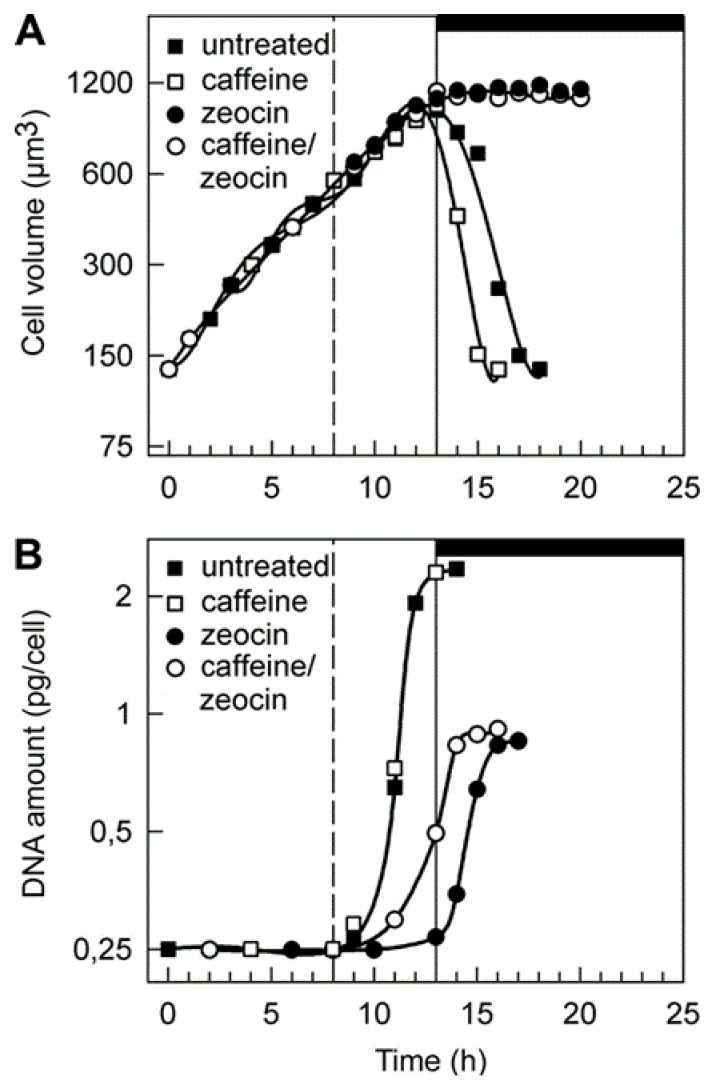
Cell growth and increase in DNA in synchronized populations of *C. reinhardtii* cells. The upper graph (**A**) depicts changes in size of cells grown in the absence of chemicals (full squares), or presence of caffeine (empty squares), zeocin (full circles) and caffeine/zeocin (empty circles). The bottom graph (**B**) shows the course of DNA replication in the absence of chemicals (full squares), or presence of caffeine (empty squares), zeocin (full circles) and caffeine/zeocin (empty circles). The time of addition of the chemicals is indicated by the dashed line, light and dark periods are marked by stripes above the panel and by the vertical solid line. Means from at least three independent experiments are presented; the standard error did not exceed 5%. Cell growth was not affected by the presence of the drugs, and DNA replication was delayed in the presence of zeocin and caffeine/zeocin.

**Figure 4:**
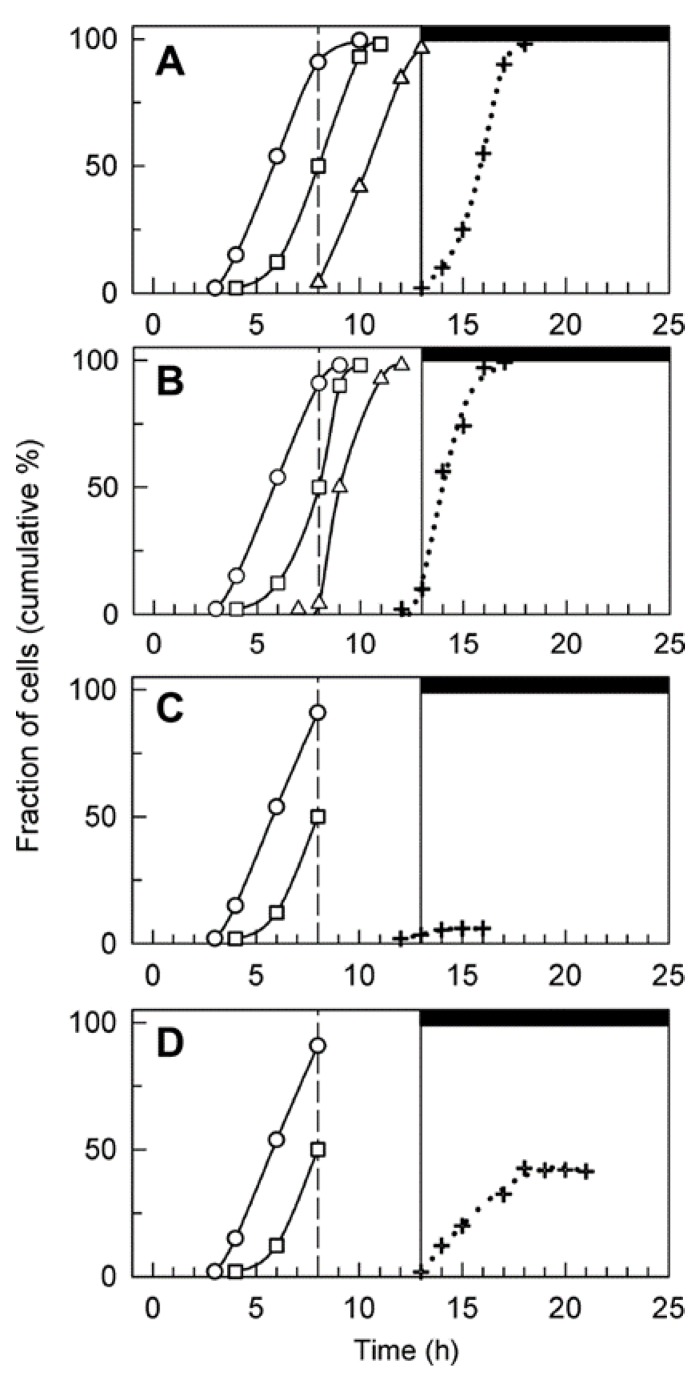
Cell cycle progression of untreated (**A**), caffeine-treated (**B**), zeocin-treated (**C**) and caffeine/zeocin-treated (**D**) synchronized populations of *C. reinhardtii* cells. The graphs show the percentages of cells that attained CP (empty symbols, solid lines) for division into two (circles), four (squares), and eight (triangles) cells, and completed cell division (crosses, dotted line). For caffeine/zeocin treated cultures, the extent of attempted aberrant division is plotted. The addition of caffeine and zeocin at the eighth hour of the cell cycle is marked by the dashed line, light and dark periods are indicated by stripes above the panel and by the vertical solid line. Means from at least four independent experiments are presented; the standard errors did not exceed 5% and are hidden within the symbols. Cell division was accelerated in the presence of caffeine and blocked in the presence of zeocin; about forty percent of the cells attempted an aberrant division in the presence of caffeine/zeocin.

The addition of the compounds did not affect cell growth, as the increase in cell size during the light period was comparable in all cultures (Figure 3A). An increase in cell volume was linked to attainment of CP, i.e., to the cell cycle entry. During the first eight hours in light, the cells sequentially attained two complete CPs (for division into two and four cells) (Figure 4) as assessed by plating the cells in darkness. However, further attainment of CPs was prevented in zeocin- and caffeine/zeocin-treated cultures (Figure 4C,D). When, for the determination of CP curves, the cells were incubated on HS medium free of drugs, the zeocin- and caffeine/zeocin-treated cultures escaped the effect of the chemicals and divided in the dark as committed. However, this rescue decreased significantly with time spent in the presence of the drugs. Cells that spent three hours or more in the presence of zeocin and caffeine/zeocin remained arrested after 30 hours. Furthermore, when the cells were plated at 20th hour of the cell cycle, i.e., after spending twelve hours in the presence of zeocin or caffeine/zeocin, their viability dropped to about 1% of the untreated cells or less than that, respectively.

### 3.3. Aberrant Cell Division in the Presence of Zeocin and Caffeine

**Figure 5:**
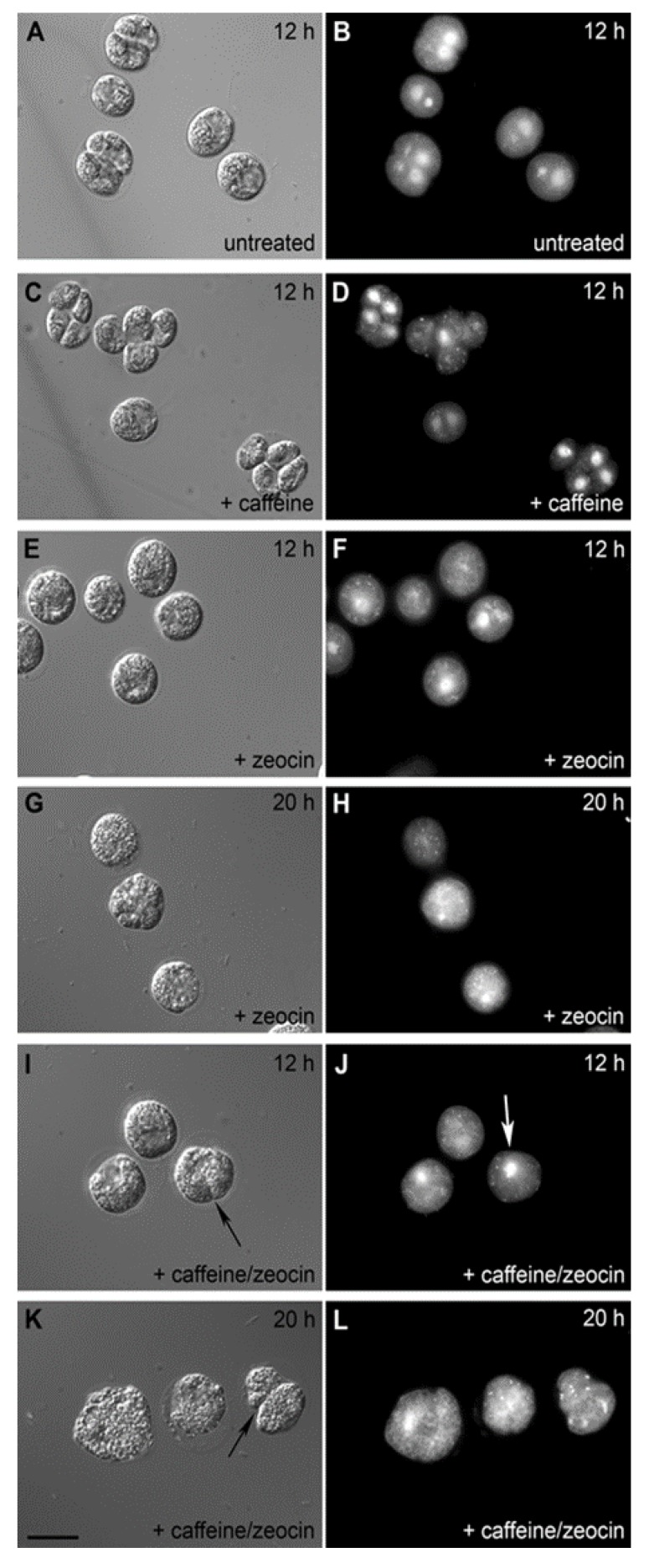
Photomicrographs of untreated (**A**,**B**), caffeine-treated (**C**,**D**), zeocin-treated (**E**–**H**) and caffeine/zeocin-treated (**I**–**L**) *C. reinhardtii* cells from the 13th hour of the synchronized cell cycle. Cells in transmitted light (A, C, E, G, I, K), fluorescence photomicrographs (B, D, F, H, J, L) of cells with nuclei stained by SYBR Green I dye. Bar = 10 µm (all photomicrographs). The untreated and caffeine-treated cells divide their nuclei and cells; zeocin and caffeine/zeocin-treated cells stay uni-nuclear, the caffeine/zeocin-treated cells attempt to proceed with aberrant cell division (black arrow) in the absence of nuclear division (white arrow).

**Figure 6:**
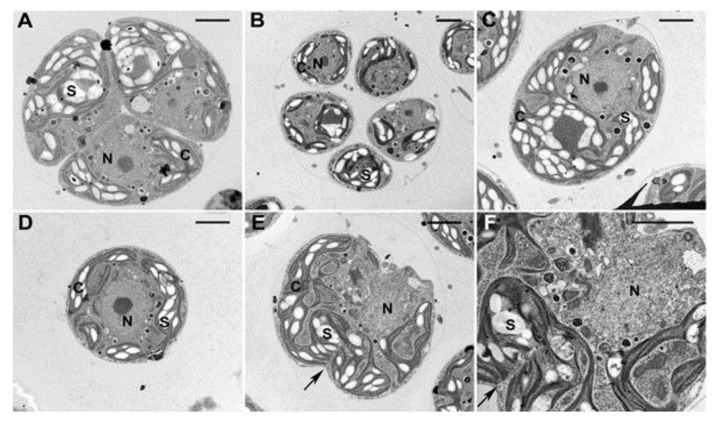
Electron microphotographs of cell divisions in untreated (**A**), caffeine-treated (**B**), zeocin-treated (**C**) and caffeine/zeocin-treated (**D**–**F**; F is an enlargement of E) *C. reinhardtii* cells. Bar = 2 µm. The untreated and caffeine-treated cells divided their nuclei (N) and chloroplasts (C) with large amounts of starch (S), zeocin and caffeine/zeocin-treated cells stay uninuclear (N), about 40% of the caffeine/zeocin-treated cells divided their chloroplasts and attempted to proceed with cell division (E, F arrow).

As discussed above, under standard conditions the vegetative reproduction of *C. reinhardtii* cells is a series of DNA replications, each followed by nuclear division—alternating S/M phase [19]. In our experiments, untreated cells started to replicate their DNA at 9 h into the cell cycle (Figure 3B); from 12 h the cells proceeded through nuclear and cellular divisions (Figure 4A, Figure 5A, Figure 6A). DNA in untreated and caffeine-treated cells increased about eight-fold, while in zeocin- and caffeine/zeocin-treated cells, it increased almost four-fold (Figure 3B). The timing of DNA replication differed between the variants, being delayed by two hours in caffeine/zeocin and by a further two hours in the zeocin-treated cultures.

Different time points of application were tested for treatments with caffeine, zeocin and their combinations. The application of chemicals at the 8th hour of the cell cycle did not affect growth but caused a cell cycle arrest. In contrast, earlier applications of the compounds affected growth as well as the cell cycle and thus were not suitable for studying the effect of compounds solely on cell cycle progression. Later applications had no effect on growth but the cell cycle block was, at least partially, abolished. Cell division was accelerated by about two hours in the presence of caffeine in comparison to untreated cells (Figure 4, compare 4A and 4B, or Figure 5A,B and Figure 5C,D or Figure 6A and Figure 6B). The zeocin-treated cells completely failed to divide their nuclei and cells (Figure 4C, Figure 5E–H, Figure 6C) although their DNA increased by almost four-fold compared to levels at the start of the cell cycle (Figure 3B). The DNA accumulation was similar in caffeine/zeocin-treated cells (Figure 3B) and about 40% of the cells attempted cell division in the absence of nuclear division, producing aberrant structures of nearly divided cells with a single nucleus (Figure 4D, Figure 5I–L, Figure 6D–F). Therefore, cells in the presence of caffeine were, at least partially, able to overcome the DNA damage checkpoint induced by the application of zeocin.

### 3.4. Elevated Kinase Activities in the Presence of Zeocin

**Figure 7:**
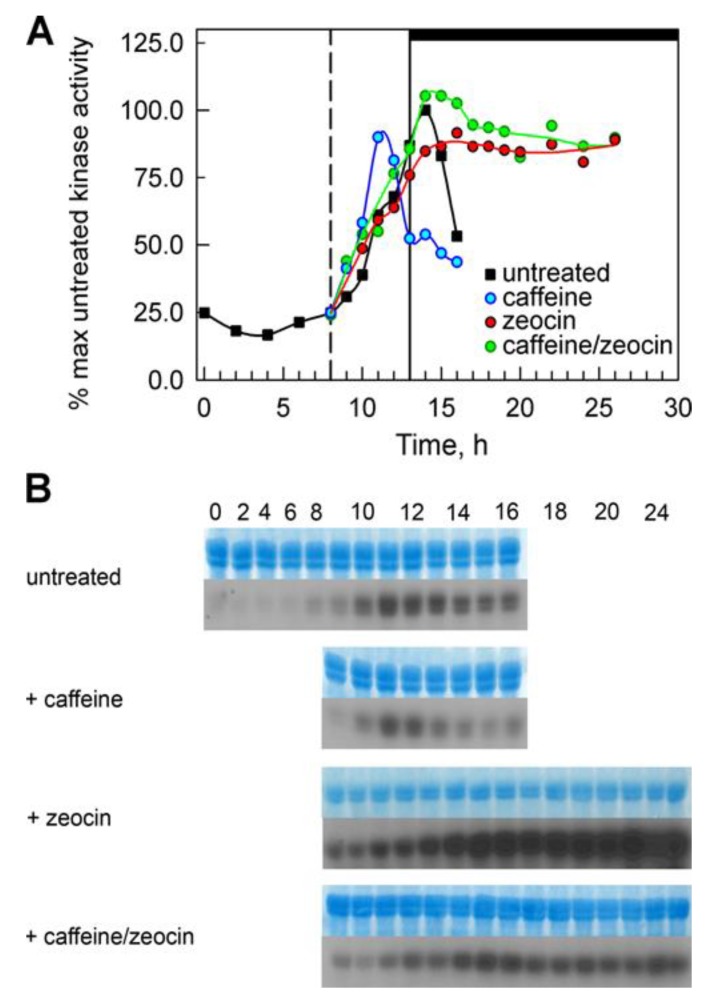
Kinase activities of CKS1-bound kinases CDKs were purified on CrCKS1 beads from cells grown in the absence or presence of caffeine, zeocin and caffeine/zeocin. Kinase activities towards histone H1 as a substrate are shown. (**A**) Means from at least four independent experiments are presented. (**B**) A representative image from one experiment is shown. Upper panel: gel stained by Coomassie Brilliant Blue for loading control, bottom panel: autoradiogram; the time of sampling in hours is depicted on the top of panels.

The lack of cell division in the presence of zeocin could have been caused by the absence of active cell cycle regulators, CDKs, or their down-regulation due to the active DNA damage checkpoint. In *C. reinhardtii*, there are two main cell cycle regulatory kinases, CDKA and CDKB with non-overlapping functions [48]. CDKA promotes entry into the cell division cycle at CP as well as DNA replication initiation [48]. CDKB is the specific mitotic kinase being essential specifically for spindle formation, nuclear division as well as re-replication [48]. The presence of DNA replication in both zeocin- and caffeine/zeocin-treated cultures suggested that the CDK-mediated initiation of DNA replication worked normally, although with a delay. In contrast, the mitotic CDK seemed to be affected. To characterize mitotic kinase activities, protein extracts were affinity purified on CrCKS1 beads [29]. Such CrCKS1-precipitable kinase activity contains complexes of both CrCDKA1 and CrCDKB1 [48]. In untreated cultures, the kinase activity bound to CKS1 beads increased after 11/12 h (start of S/M phase), reached a peak at 14 h and started to decrease thereafter. In caffeine-treated cultures, the kinase activity increased and reached a peak about three hours sooner compared to untreated cultures (Figure 7), which was in agreement with earlier nuclear and cellular divisions in these cultures. In the zeocin-treated cultures, the kinase activities remained comparable to the untreated control until 13 h; thereafter the kinase activities were maintained at high levels until the end of the experiment (Figure 7). The kinase activities showed similar kinetics in the caffeine/zeocin-treated cultures. The increase in kinase activity started at the same time as in untreated cultures. However, in contrast to the untreated cultures, it stayed elevated for the rest of the experiment (Figure 7).

## 4. Discussion

The presence of DNA damage initiates a signaling cascade, DNA damage checkpoint, which will lead to an activation of DNA repair as well as to a delay in cell cycle progression in order to buy time for the repair [3]. In agreement with this, synchronized cultures of *C. reinhardtii* cultivated in the presence of zeocin blocked nuclear and cell divisions (Figure 4C, Figure 5E–H, Figure 6C). This resembled the previously established DNA damage checkpoint induced by UV irradiation, causing a nuclear and cell division delay by about five hours [49]. The cell cycle delay was crucial for cell survival as its absence in cells of a checkpoint mutant (uvs11) led to premature cell division, formation of microcolonies and cell death [49]. Similarly, simultaneous application of zeocin and caffeine led to increased sensitivity to zeocin and premature cell death (Figure 2), probably due to accelerated cell division entry (Figure 4, Figure 6). This suggests the delay in cell division was required for improved cell survival, possibly due to providing a time window for DNA damage repair. Although both UV irradiation and application of zeocin caused a delay in cell division, the two checkpoints differed in several aspects. The UV irradiation did not affect the timing of DNA replication, which occurred similarly in both the wild type and uvs11 mutant in the presence or absence of the UV treatment [49]. This was in contrast to about a four-hour delay in DNA replication caused by application of zeocin or about a two hour delay caused by a combination of zeocin and caffeine (Figure 3). Furthermore, the UV irradiation-induced DNA checkpoint lasted for about five hours, after which cell division resumed completely [49]. On the other hand, cells in the presence of zeocin never recovered cell division (Figure 4) and even cells with diminished DNA damage checkpoint in the presence of caffeine/zeocin attempted the cell division only partially (Figure 4, Figure 6). Finally, the UV irradiation DNA checkpoint decreased CDK activity [49] whilst CDK activity was increased in the presence of zeocin (Figure 7). These differences could point to the existence of two types of DNA damage checkpoints in *C. reinhardtii*: 1) a short-term G2/M checkpoint with unaffected DNA replication timing and decreased CDK activity, and 2) a prolonged checkpoint encompassing both DNA replication delay (S phase checkpoint) and G2/M block accompanied with high CDK activity. It would be interesting to gain insight into the similarities and/or differences in the molecular mechanisms underpinning these two checkpoints. The mutation-abrogating UV irradiation induced checkpoint has been described [49] but the causative genes have not yet been identified.

Interestingly, the two DNA damaging conditions differ in their sensitivity to caffeine. Trimethylxanthine caffeine uncouples mitosis from the completion of DNA replication [50,51], accelerates mitosis [45,52,53], and overrides DNA replication and/or DNA damage checkpoints in various systems [50,54,55,56,57,58] due to specific inhibition of ATM/ATR kinases [45,46,47]. A combination of the application of caffeine with UV irradiation improved survival of both wild type and recombination-proficient UV sensitive mutants [59]. This suggested that DNA repair of UV irradiation-caused adducts might occur not only by the well-established dark reactions of nucleotide-excision repair but also by recombination [60]. In contrast, the combination of zeocin and caffeine had a detrimental effect on cell survival (Figure 2). This would indicate that caffeine repressible compound(s), possibly ATM/ATR kinase(s) as in other systems [45,46,47], are required for proper repair of double stranded breaks.

Caffeine simultaneously present with zeocin had two major effects on the observed DNA damage checkpoint(s): 1) it partially overrode the DNA replication checkpoint by accelerating DNA replication, 2) it partially overrode the G2/M checkpoint and pushed the cells to premature cell division in the absence of nuclear division (Figure 6E,F). This is in contrast to higher plants where caffeine was shown to override DNA damage but not the DNA replication checkpoint [55,56]. The effect of caffeine on *C. reinhardtii* was, in both cases, only partial and its presence was unable to induce nuclear division (Figure 6E,F). This could point either to a low concentration of caffeine being used or to the existence of an alternative DNA checkpoint pathway that is either insensitive to caffeine or requires its higher concentration. The existence of an additional caffeine-insensitive pathway was indicated by experiments in another green alga, *Desmodesmus quadricauda.* The application of caffeine to synchronized cultures also partially overrode DNA damage checkpoint induced by the presence of zeocin from the beginning of the cell cycle. If zeocin was applied later, during G2/M phase, the cells did not experience DNA damage checkpoint fully and about 60% of the synchronized population proceeded to mitosis. The proportion of the population undergoing nuclear (and cell) division(s) could be further increased by application of caffeine [61]. This implied the existence of two DNA damage pathways, one insensitive to caffeine and present preferentially prior to G2 phase and the other, a caffeine sensitive one.

The high CDK activity in the presence of zeocin seemed controversial and it was in striking contrast to the lowered kinase activity during the UV irradiation-induced DNA damage checkpoint [49]. One explanation would be that active CDK is specifically required for repair of double-stranded breaks as in mammals [62,63], in budding yeast [64], and in higher plants where CDKB1-CYCB1 complexes mediate homologous recombination [65,66]. At the same time, the phenotypes of zeocin and caffeine/zeocin-treated cells resembled phenotypes of arrested temperature sensitive cell division cycle mutants of key cell cycle regulatory kinases isolated and described by F. Cross and his colleagues [48,67]. Namely, that zeocin treated cells resembled arrested cells of the *cdka-1* mutant with delayed DNA replication and the absence of notches of attempted cell division but did not fit the mutant phenotype due to high kinase activity [48]. In contrast, caffeine/zeocin treated cells resembled those of cdkb1-1 (and cycb1-1) mutants with delayed DNA replication, high CDK activity and notches of attempted cell division [48,67]. The similarity of phenotypes might imply involvement of both CDKs in the DNA damage checkpoint, with a specific role of the CDKB1-cyclin B complex in the caffeine-insensitive pathway. This would fit with the situation in higher plants where degradation of cyclin B2 is involved in the caffeine-insensitive DNA replication checkpoint in S phase [56] and degradation of CDKB2 is a part of the response to double-stranded breaks [16]. Thus, the CDKB-cyclin B complex might, in *C. reinhardtii,* be targeted by the DNA damage checkpoint. However, to test such an hypothesis, further experiments on the effect of DNA damage in the mutants are required.
